# Chronic E-Cigarette Aerosol Inhalation Alters the Immune State of the Lungs and Increases ACE2 Expression, Raising Concern for Altered Response and Susceptibility to SARS-CoV-2

**DOI:** 10.3389/fphys.2021.649604

**Published:** 2021-05-31

**Authors:** Jorge A. Masso-Silva, Alexander Moshensky, John Shin, Jarod Olay, Sedtavut Nilaad, Ira Advani, Christine M. Bojanowski, Shane Crotty, Wei Tse Li, Weg M. Ongkeko, Sunit Singla, Laura E. Crotty Alexander

**Affiliations:** ^1^Pulmonary Critical Care Section, VA San Diego Healthcare System, La Jolla, CA, United States; ^2^Division of Pulmonary, Critical Care and Sleep Medicine, Department of Medicine, University of California, San Diego (UCSD), La Jolla, La Jolla, CA, United States; ^3^Division of Pulmonary Critical Care, Department of Medicine, Tulane University, New Orleans, LA, United States; ^4^Department of Medicine, La Jolla Institute of Allergy and Immunology, La Jolla, CA, United States; ^5^Department of Otolaryngology-Head and Neck Surgery, UCSD, La Jolla, CA, United States; ^6^Division of Pulmonary Critical Care, Department of Medicine, University of Illinois, Chicago, IL, United States

**Keywords:** e-cigarette, vaping, ACE2, COVID-19, immunomodulation, SARS-CoV-2, RNAseq, lipidomics

## Abstract

Conventional smoking is known to both increase susceptibility to infection and drive inflammation within the lungs. Recently, smokers have been found to be at higher risk of developing severe forms of coronavirus disease 2019 (COVID-19). E-cigarette aerosol inhalation (vaping) has been associated with several inflammatory lung disorders, including the recent e-cigarette or vaping product use-associated lung injury (EVALI) epidemic, and recent studies have suggested that vaping alters host susceptibility to pathogens such as severe acute respiratory syndrome coronavirus 2 (SARS-CoV-2). To assess the impact of vaping on lung inflammatory pathways, including the angiotensin-converting enzyme 2 (ACE2) receptor known to be involved in SARS-CoV-2 infection, mice were exposed to e-cigarette aerosols for 60 min daily for 1–6 months and underwent gene expression analysis. Hierarchical clustering revealed extensive gene expression changes occurred in the lungs of both inbred C57BL/6 mice and outbred CD1 mice, with 2,933 gene expression changes in C57BL/6 mice, and 2,818 gene expression changes in CD1 mice (>abs 1.25-fold change). Particularly, large reductions in IgA and CD4 were identified, indicating impairment of host responses to pathogens *via* reductions in immunoglobulins and CD4 T cells. CD177, facmr, tlr9, fcgr1, and ccr2 were also reduced, consistent with diminished host defenses *via* decreased neutrophils and/or monocytes in the lungs. Gene set enrichment (GSE) plots demonstrated upregulation of gene expression related to cell activation specifically in neutrophils. As neutrophils are a potential driver of acute lung injury in COVID-19, increased neutrophil activation in the lungs suggests that vapers are at higher risk of developing more severe forms of COVID-19. The receptor through which SARS-CoV-2 infects host cells, ACE2, was found to have moderate upregulation in mice exposed to unflavored vape pens, and further upregulation (six-fold) with JUUL mint aerosol exposure. No changes were found in mice exposed to unflavored Mod device-generated aerosols. These findings suggest that specific vaping devices and components of e-liquids have an effect on ACE2 expression, thus potentially increasing susceptibility to SARS-CoV-2. In addition, exposure to e-cigarette aerosols both with and without nicotine led to alterations in eicosanoid lipid profiles within the BAL. These data demonstrate that chronic, daily inhalation of e-cigarette aerosols fundamentally alters the inflammatory and immune state of the lungs. Thus, e-cigarette vapers may be at higher risk of developing infections and inflammatory disorders of the lungs.

## Introduction

Electronic cigarettes (e-cigarette) arose as a safer way to deliver nicotine than conventional cigarettes, with the ultimate goal of helping smokers quit tobacco (Bozier et al., 2020). E-cigarette use has increased significantly over the last 10 years (Fadus et al., 2019), despite of the lack of sufficient data about the effects of these devices and its content being delivered (Crotty Alexander L. et al., 2015; Crotty Alexander L. E. et al., 2015). This is due in part to the advertisement of these drug delivery devices as safer and containing less toxins than conventional cigarettes (Bozier et al., 2020). Unfortunately, e-cigarettes have not been found to be successful smoking cessation tools (Bullen et al., 2013; Glantz and Bareham, 2018), and in fact many users who tried using them for cessation end up as dual users of both conventional tobacco and e-cigarettes (Stratton et al., 2018). In addition, the use of e-cigarettes may contribute to relapse of smoking in ex-smokers (Gomajee et al., 2019; McMillen et al., 2019). More worrisome is the growing population of e-cigarette users whom are never smokers, including large numbers of adolescents and young adults (Berry et al., 2019; Fadus et al., 2019). Therefore, from a socioeconomical perspective to a self-awareness of the effect of these devices, it is crucial to understand the implications of their use in health.

Vaping of e-cigarettes has been associated with numerous inflammatory disorders of the lungs, including hypersensitivity pneumonitis, lipoid pneumonia, and eosinophilic pneumonia, demonstrating that the inhalation of chemicals within e-cigarette aerosols can alter the inflammatory state of the lung (Sommerfeld et al., 2018; Viswam et al., 2018; Arter et al., 2019). In 2019, an epidemic of lung injuries associated with vaping occurred in the United States (Chatham-Stephens et al., 2019). The primary clinical diagnosis in these patients was acute respiratory distress syndrome (ARDS) or acute lung injury (ALI). Almost 3,000 e-cigarette or vaping device-associated lung injury (EVALI) cases had been reported to the Centers for Disease Control and Prevention by late November 2019, with 68 deaths confirmed by February 2020 (Chatham-Stephens et al., 2019). Since then, data on EVALI has not been updated by the CDC, due to the coronavirus disease 2019 (COVID-19) pandemic.

Since there are numerous e-cigarette devices and a thousands of varieties of e-liquids (chemicals in liquid form that are aerosolized by e-devices), the specific chemicals responsible for the lung injuries have not yet been confirmed, but vitamin E acetate and tetrahydrocannabinol (THC) have been closely tied to this particular vaping induced lung disease (Blount et al., 2020; Crotty Alexander et al., 2020).

Outside of directly causing lung diseases, including EVALI, e-cigarette vaping may disrupt sleep (Boddu et al., 2019), induce fibrosis in the heart, liver, and kidneys (Crotty Alexander et al., 2018), alter neutrophil function and host defenses (Corriden et al., 2020), increase systemic inflammation (Crotty Alexander et al., 2018), and increase susceptibility to infections (Sussan et al., 2015; Madison et al., 2019; Corriden et al., 2020). Researchers have shown increased severity of influenza pneumonia in murine models of e-cigarette exposure (Madison et al., 2019), which raises the question of whether other viral lung infections might also be impacted by e-cigarette exposure. Because of a lack of medical coding for e-cigarette and vaping device use, and because healthcare professionals do not consistently ask patients about vaping and dabbing, using medical records to answer the critical question of how vaping impacts susceptibility to infection with severe acute respiratory syndrome coronavirus 2 (SARS-CoV-2) and development of COVID-19 will be difficult. Thus, we hypothesized that vaping may alter inflammatory responses in the lungs, which may relate to changes that are known to increase susceptibility to SARS-CoV-2 infection, which could explain why younger patients can develop COVID-19 even with no apparent pre-existing medical conditions or alternatively exacerbate the diseases if pre-existing conditions are present.

The aim of this study was to broadly assess the effects of e-cigarette aerosols on lung inflammatory responses and resting immune state *via* unbiased methodologies. We assessed overall gene expression changes in the lungs upon chronic inhalation exposure to e-cigarette aerosols in two disparate mouse backgrounds—the inbred C57BL/6 strain and outbred CD1 strain. We assessed changes in inflammatory factors and host defenses due to inhalation of aerosolized vehicle components alone, versus with the addition of nicotine. Because it is unknown whether vaping predisposes to higher severity of COVID-19, we specifically assessed for changes in inflammation in the lung, including activation of neutrophils, which are considered a primary driver of ARDS pathogenesis. It is also unknown whether vaping, like conventional cigarette smoking, increases susceptibility to infection with SARS-CoV-2, therefore we specifically assessed for angiotensin-converting enzyme 2 (ACE2) expression, which is crucial for the infectivity of SARS-CoV-2, finding that indeed e-cigarette use may increase ACE2 expression and that flavorant chemicals within e-liquids might drive ACE2 expression in particular.

## Materials and Methods

### E-Cigarettes

Three types of vaping devices were used for these studies: vape pens, box Mods, and pod devices (JUUL). Kanger Mini-protank glassomizers with 1.5 Ohm coils, attached to Kanger eVOD variable voltage 1000 mAh batteries set at 4.8 Volts, were the vape pen devices used for these studies. The box Mod used was a Kanger base with direct electricity from the wall and glass and metal Kanger protanks. For vape pens and box Mods, chemical components of e-liquids were purchased from Sigma and mixed in the lab prior to filling the tanks. For vape pen exposures, propylene glycol (PG) was mixed 1:1 with glycerin (Gly) to create a 50:50 solution with nicotine at 24 mg/ml. Sixty-minute vape pen exposures corresponded with plasma cotinine (the primary metabolite of nicotine) levels of 269 ± 17 ng/ml. For box Mod exposures, e-liquids were mixed at 70:30 PG:Gly with and without 6 mg/ml nicotine, to produce both vehicle e-cigarette aerosol without nicotine and regular e-cigarette aerosol with nicotine (EV). Sixty-minute box Mod exposures with e-liquid containing nicotine corresponded with plasma cotinine levels of 253 ± 21 ng/ml. For the pod devices, we used the two most popular JUUL flavors in 2018–2019, mint and mango. JUUL batteries and pods were purchased directly from the manufacturer in bulk, and all lot numbers were recorded. E-liquids from JUUL pods have 30:70 PG:Gly, nicotine salts at 59 mg/ml, benzoic acid, and flavorant chemicals (Talih et al., 2019). Twenty-minute JUUL mint exposures corresponded with plasma cotinine levels of 194 ± 14 ng/ml, and JUUL mango with 216 ± 38 ng/ml.

### E-Cigarette Aerosol Exposures in Mice

Inbred C57BL/6 and outbred CD1 mice were purchased from Envigo at 6–8 weeks of age. Two strains of mice with different genetic backgrounds, which have different susceptibilities to inflammatory and infectious challenges, were utilized to increase the likelihood of biological relevance to humans by identifying vaping induced changes which occur in both, and thus are not limited to one specific genotype or phenotype. Studies were first run in female mice (*n* = 6 per group) of both strains, with further studies conducted in male C57BL/6 mice. All animal studies were conducted with prior approval of both UCSD and VA Institutional Animal Care and Use Committees (IACUC). Mice were randomized prior to exposures. For vape pen and box Mod exposures, mice were placed into the Scireq inExpose whole-body exposure system for 60 min once daily for 3–6 months. JUUL exposures were done in 20-min blocks three times daily for 1 month. As previously described, e-cigarettes were activated and flow generated *via* application of negative pressure every 20 s, with puff duration of 4 s across all devices (Alasmari et al., 2017, 2019; Crotty Alexander et al., 2018; Corriden et al., 2020). Mice were recovered in prewarmed cages for 20 min after each exposure.

### Bronchoalveolar Lavage and Lung Tissue Harvest

After the final e-cigarette aerosol exposure, mice were anesthetized and sedated with 100 mg/kg ketamine and 10 mg/kg xylazine and underwent tracheostomy. Bronchoalveolar lavage (BAL) was performed with 500 μl cold 1× PBS three times, with pooling of the recovered fluid. BAL was centrifuged at 1,800 μrpm at 4°C for 8 min and supernatant aliquoted and snap frozen prior to transfer to the lipidomics core at UCSD. Right lung lobes were harvested, placed into RLT buffer (Qiagen), snap frozen, and stored at -80°C prior to RNA extraction.

### Lipidomics

Bronchoalveolar lavage samples were submitted to the LIPID MAPS Lipidomics Core at UCSD and underwent broad profiling and quantitative analysis of eicosanoids using liquid-chromatography mass spectrometry (LC-MS/MS) platforms. In brief, 157 eicosanoids and *N*-acylethanolamines were analyzed by UPLC-MS/MS and the steady-state levels fully quantitated by comparison with authentic standards. BAL (30–200 μL) was introduced, along with 1,000 μL of internal standard mix, and extraction was performed with SPE using strata-x polymeric reversed-phase columns (8B-S100-UBJ Phenomenex). Samples were injected into UPLC (Acquity UPLC System, Waters) followed by analysis by mass spectrometry (Sciex 6500 Qtrap). Data were normalized to volume (pmol/ml), and two-way ANOVA with Tukey multiple comparisons test was used to assess differences in lipids across the three exposure groups. Data represents the dynamic balance between synthesis and secretion, and catabolism and clearance, of eicosanoids in the airways of mice exposed to e-cigarette aerosols with and without nicotine.

### RNAseq

Total RNA from whole lung was isolated (RNeasy mini kit, QIAGEN) and submitted to the UCSD IGM Genomics Core for processing, quality control checks, creation of libraries, and sequencing (HiSeq4000).

### qPCR

Total RNA was isolated from whole lung (RNeasy mini kit, Qiagen), converted to cDNA (Applied Biosystems^TM^ High Capacity cDNA Reverse Transcription Kit, Thermo Fisher), and underwent qPCR with Taqman Gene Expression Assays for mouse ACE2 (Mm01159006_m1) and mouse GAPDH (Mm99999915_g1) primers, oligonucleotides, FAM dye, with TaqMan^®^ Fast Advanced Master Mix (Thermo Fisher), per manufacturer’s RT-PCR instructions in a 384-well plate RT-PCR thermal cycler (Biorad). Data were analyzed by one-way ANOVA with Sidak’s multiple comparisons test.

### RNAseq Statistical Analysis

Sequencing data underwent DESeq2 normalization (Chen et al., 2018) followed by analysis at the La Jolla Institute of Allergy and Immunology. An unbiased approach was used to identify e-cigarette aerosol inhalation-dependent gene expression changes by taking all lung samples from both strains of mice (C57BL/6 and CD1) and filtering for the largest gene expression changes upon e-cigarette exposure (genes showing a fold change of 1.25 and adjusted *p*-value of less than 0.1), followed by hierarchical clustering to create a heat map. A volcano plot was used to highlight the greatest changes. Bubble GUM (GSEA unlimited map) charts were drawn using the Benjamini–Yekutieli *p*-value correction method, using controls even if tests were dependent. The gene sets were filtered based on Benjamini–Yekutieli Padj (FDR) < 0.25 (allowing only for 25% false positives). For cell-specific heat maps, transcripts per kilobase million (TMP) were calculated for the genes and no clustering was performed to keep the cell-specific gene sets intact.

### Calculation of Gene Fold Changes for qPCR Analysis

Gene expression fold change was calculated for ACE2 and ACE2 interactor genes in lung tissue from mice exposed to EV vs. air control mice. Fold change was calculated as the median of expression in the EV-exposed cohort divided by the median of expression in the normal cohort. A list of interactor genes was obtained from Pathways Commons^1^, an aggregator of interactions drawn from major pathway databases.

### Correlation of ACE2 Expression With Immune Infiltration and Immune-Associated Pathways

Estimates of abundance of immune cell populations were inferred using the program Cibersortx^2^ (Chen et al., 2018), which deconvolutes bulk RNA sequencing data to derive the infiltration levels of 22 different immune cell types. These infiltration levels are correlated to ACE2 expression using the Spearman correlation test (*p* < 0.05). In addition, ACE2 expression was also correlated with the upregulation or downregulation of immune-associated pathways using gene set enrichment analysis (GSEA, *p* < 0.05). Pathways were obtained from the Pathways Interaction database (PID) and the Reactome database.

## Results

### Exposure to E-Cigarette Aerosols Induces Profound Gene Expression Changes in Key Immune and Inflammatory Pathways in the Lungs of Two Disparate Mouse Genetic Backgrounds

**FIGURE 1:**
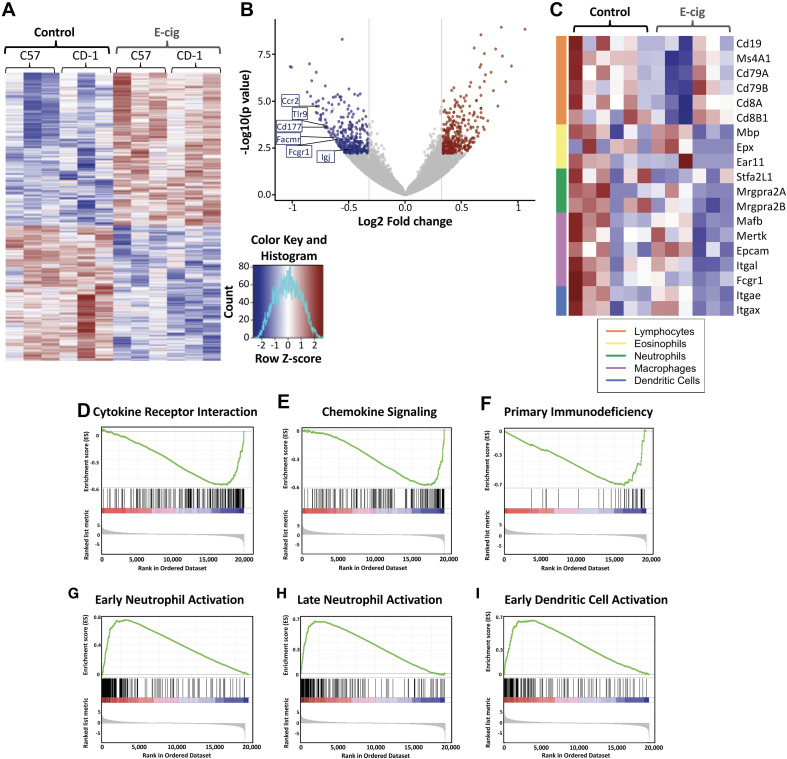
Exposure to e-cigarette aerosol induces profound effects in overall inflammatory responses in two different mouse genetic backgrounds. Transcriptomic analysis of lung tissue performed by RNAseq from CD1 and C57BL/6 female mice exposed daily to e-cigarette aerosol generated by Vape pens with 50:50 PG:Gly and 24 mg/ml nicotine for 3 and 6 months, respectively, detected 2,818 (CD1) and 2,933 (C57BL/6) gene expression changes. **(A)** Heat map of differentially expressed (fold change of 1.25 and adjusted *p*-value less than 0.1) genes with hierarchical clustering revealed many genes with expression changes in both strains of mice after e-cigarette exposure as compared to Air controls. **(B)** Examination of gene expression changes above 1.5-fold (vertical lines) by volcano plot revealed several genes of interest. Igj was greatly reduced, indicating that e-cigs substantially impair lung IgA expression. CD177, Facmr, Tlr9, Fcgr1, and Ccr2 were reduced, indicating a further loss of host defenses. **(C)** Assessing immune cellspecific gene expression changes in the lung tissue demonstrated alterations across lymphocytes, eosinophils, neutrophils, macrophages and dendritic cells. Enrichment plots demonstrated profound downregulation of gene expression in broad immune signaling **(D,E)** pathways, important for **(D)** cytokine receptor interactions and **(E)** chemokine signaling. **(F)** Downregulation across genes of importance in primary immunodeficiency was seen, while patterns of gene upregulation were detected in innate immune cells: **(G)** Early neutrophil activation and **(H)** Late neutrophil activation, and cells that act as the intersection between innate immune and adaptive cells – antigen presenting cells: **(I)** Early dendritic cell activation. Data is representative of 6 mice per group, with total lung RNA from pairs of mice pooled, giving three data points per group. The enrichment score is shown as a green line **(D–I)**, which reflects the degree to which a gene set (the barcode where each black line is a gene in the gene set) is overrepresented at the top or bottom of a ranked list of genes (the heatmap axis – red/blue/white).

Hierarchical clustering revealed extensive gene expression changes occurred in the lungs of both inbred C57BL/6 mice and outbred CD1 mice whom inhaled e-cigarette aerosols daily for 3–6 months, with 2,933 gene expression changes in C57BL/6 mice, and 2,818 gene expression changes in CD1 mice (>abs 1.25-fold change; Figure 1A). Examination of the largest changes by volcano plot revealed several genes of interest (Figure 1B). Expression of IgA was greatly reduced in CD1 mice, indicating that e-cigarette exposure may lead to altered IgA levels in the lungs. CD177, facmr, tlr9, fcgr1, and ccr2 were all reduced, suggesting diminished host defenses *via* decreased neutrophils and/or monocytes in the lungs upon e-cigarette aerosol exposure. In addition, assessing immune cell-specific gene expression changes in the lung tissue demonstrated alterations across lymphocytes, eosinophils, neutrophils, macrophages, and dendritic cells (Figure 1C). CD4 was also reduced in CD1 but not C57BL/6 mice, suggesting a strain-specific reduction of CD4^+^ T cells from the lungs of CD1 mice upon e-cigarette aerosol exposure, again indicating immunomodulation that may increase the risk of infection and dysregulate the immune response to an infection. The most upregulated gene in both strains of mice was Krt83, an uncommon keratin gene expressed in epithelial cells. Krt8 expression has been found to be increased in some carcinomas (Chu and Weiss, 2002; Gires et al., 2006) and promotes tumor progression and metastases in gastric carcinoma in particular (Fang et al., 2017).

KEGG enrichment plots demonstrated profound downregulation of gene expression in key immune and inflammatory pathways in lungs of e-cigarette-exposed mice versus air controls (Figures 1D–I). Enrichment plots contain KEGG profiles of the running enrichment scores (ES) and positions of gene set members on the rank-ordered list in GSE. Cytokine receptor interaction signatures were downregulated in e-cigarette lungs versus air controls (ES -0.571, NES -2.32, *p* and *q*s = 0; Figure 1D). Notable cytokines represented in this pathway include IL-1, IL-2, IL-6, TNF, interferon gamma (IFNγ), and TGFβ. Chemokine signaling pathway signatures were downregulated in e-cigarette lungs (ES -0.585, NES -2.32, *p* and *q*s = 0; Figure 1E). Notable members of this pathway include β-arrestin, G-protein-coupled receptors, PI3K, JAK/STAT, ERK1/2, and IKK/IκB/NFκB). Genes associated with primary immunodeficiency were downregulated in e-cigarette lungs (ES -0.717, NES -2.16, *p* and *q*s = 0; Figure 1F). Genes in this pathway are specific for lymphoid lineages with members including CD3δ/ε, CD8α, CD45, IKKγ, CD40/CD40L, RAG1/2, IL1Rα, Igα, BTK, λ5, and RFX5/AP/ANK. Moreover, we also observed upregulation of gene expression related to cell activation specifically in neutrophils (Figures 1G,H) and dendritic cells (ES 0.654, NES 2.41, *p* and *q*s = 0; Figure 1I). With early (9 h; Figure 1G) neutrophil stimulation and (B) late (24 h; Figure 1H) neutrophil stimulation interaction signatures increased in e-cigarette-exposed mouse lungs versus air controls (ES 0.735 and 0.683, respectively; NES 2.75 and 2.58, respectively; *p* and *q*s = 0). Gene members of the neutrophil activation pathways include CXCR2, Stfa2l1, Csf3r, and Chi3l1, while early dendritic cell activation members include MAPK, NLRs, JAK/STAT, and TNF. These data suggest that e-cigarette aerosol exposure leads to immunomodulation of host defenses, suppressing and inducing key immune pathways (innate and adaptive) affecting the homeostatic state of the lungs.

### Inhalation of E-Cigarette Aerosols Diminished Eicosanoid Lipids Within the Airways

**FIGURE 2:**
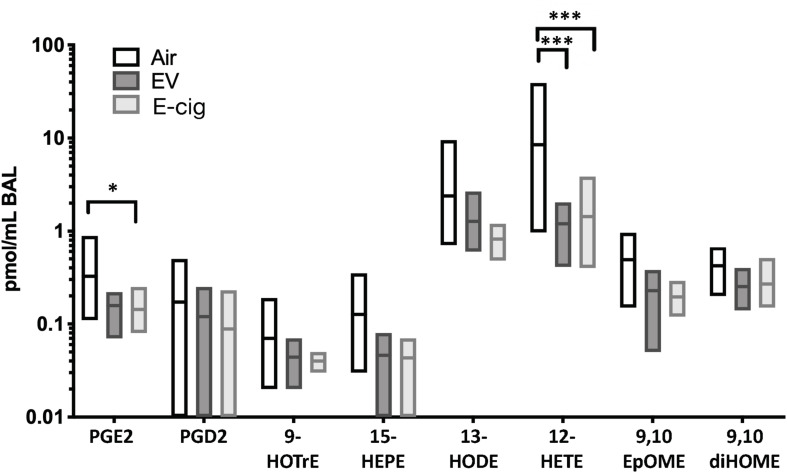
Eicosanoid lipid profiles within BAL are modestly altered by both e-cigarette vehicle and nicotine-containing (E-cig) aerosol exposures. C57BL/6 female mice were exposed for 1 h daily to ecigarette aerosols generated with e-liquid containing 70:30 PG:Gly, with (E-cig) and without 6 mg/mL nicotine (Vehicle), generated from box Mods, or Air only, for 6 months. BAL underwent broad profiling and quantitative analysis of eicosanoids using liquid-chromatography mass spectrometry (LC-MS/MS). Mice exposed to Vehicle aerosols had decreased PGE2, which is produced in airway epithelium and is a bronchodilator. Both Vehicle (no nicotine) and E-cig (with nicotine) exposed mice had decreased 12-HETE in the BAL (*p* < 0.001), suggesting changes induced by non-nicotine chemicals within aerosols. Overall, eicosanoid levels trended lower in the BAL of both Vehicle and E-cig groups relative to Air controls, suggesting broad suppression of eicosanoid production or release. Data are representative of 6 mice per group. *p < 0.05, ***p < 0.001.

E-cigarette exposure led to decreased of two main eicosanoid lipids, including prostaglandin E2 (PGE2; *p* < 0.05) and 12-hydroxyeicosatetraenoic acid (12-HETE; *p* < 0.001), which suggests dysregulation of inflammatory pathways and host defenses (Figure 2). The non-nicotine chemicals (vehicles: PG and Gly) within the aerosols appeared to be the drivers behind these subtle changes, as vehicle mice had equal to greater changes as compared with EV with nicotine. Therefore, besides the variety of inflammatory proteins altered due to e-cigarette aerosol inhalation, there are also changes in important lipids involved in inflammation.

### ACE2 Expression Is Upregulated by Exposure to E-Cigarette Aerosol

**FIGURE 3:**
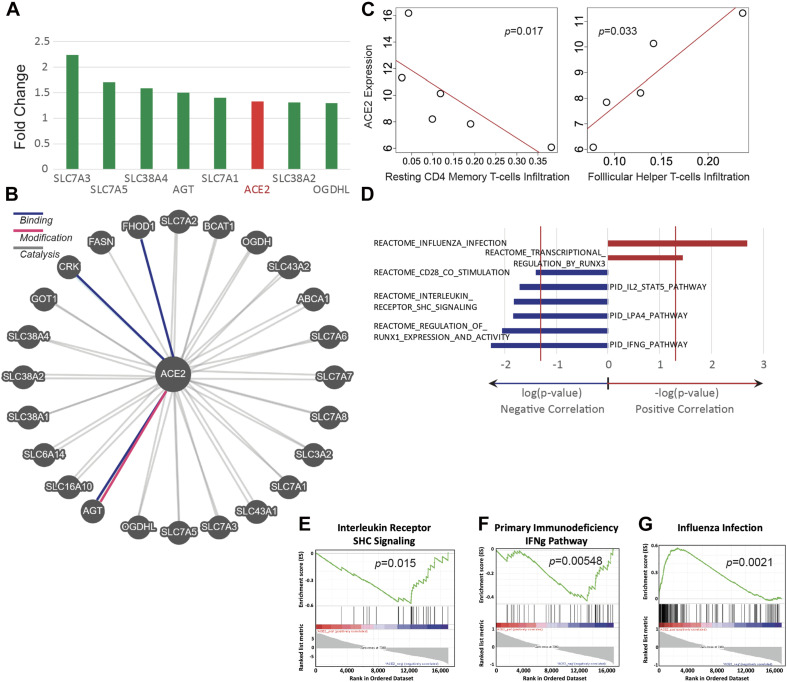
ACE2 expression is upregulated in E-cig exposed mice and correlates with immune status. Transcriptomic data from the lungs of CD1 and C57BL/6 female mice, exposed to aerosols generated from Vape pens with 50:50 PG:Gly and 24 mg/ml nicotine for 3 and 6 months, respectively, was analyzed for ACE2 expression and assessed for correlation with immune pathways. **(A)** Changes in ACE2 and ACE2-associated gene expression in the lungs of E-cig exposed mice relative to Air control mice. **(B)** Schematic of genes associated with ACE2. **(C)** Scatter plots of ACE2 expression demonstrate correlations with immune cell infiltration levels (*y*-axis plots gene expression values in read count per million reads; *x*-axis plots the fraction of immune cells within the lung tissue that are of the specific cell type indicated). **(D)** Correlations between ACE2 expression and immunity-associated pathways. The vertical red lines represent significance cutoff at *p* = 0.05. Individual GSEA plots demonstrate correlations between ACE2 expression and **(E)** interleukin receptor signaling, **(F)** the IFNg pathway in primary immunodeficiency, and **(G)** immune pathways activated during infection with influenza. Data is representative of six mice per group, with pooling of RNA from pairs of mice prior to transcriptomics.

ACE2 was found to be upregulated by 33% in mice exposed to e-cigarette aerosols generated from vape pens (Figure 3A). The expression of genes that are associated with ACE2, including angiotensinogen (AGT) and SLC7 members, were also upregulated after exposure to e-cigarette aerosols (Figures 3A,B). Angiotensinogen is the precursor of angiotensin II, the substrate of ACE2, while SLC7 members have been documented to interact with ACE2 and are considered part of the ACE2 gene network (Dai et al., 2020; Soule et al., 2020; The Lancet Respiratory Medicine, 2020). SLC proteins can physically bind to ACE2 and may affect viral entry mechanisms (The Lancet Respiratory Medicine, 2020). Thus, ACE2, a currently relevant membrane-bound enzyme (and ACE2-related genes) in the context of SARS-CoV-2 infection is upregulated when mice are exposed to e-cigarette aerosols, and genes interacting with ACE2 are also upregulated by these inhalants.

### ACE2 Expression Is Associated With Lung Immune Cell Levels and Immune-Associated Pathways

The abundance of immune cell types was assessed by deconvolving bulk RNA-sequencing data and correlated abundances to ACE2 expression. ACE2 upregulation is significantly correlated with a lower abundance of resting CD4^+^ memory T cells but with a higher abundance of follicular helper T cells (Spearman, *p* < 0.05) (Figure 3C). In addition, we found that e-cigarette-induced upregulation of ACE2 is correlated with the downregulation of several immune-associated pathways, including interleukin receptor signaling, IFNγ signaling, and CD28 co-stimulation (Figures 3D–G). Interestingly, ACE2 upregulation in e-cigarette aerosol-exposed mice is associated with increased expression of genes associated with influenza infection and transcriptional regulation by runx3, a key regulator of tissue-resident memory CD8^+^ T cell differentiation and homeostasis (Figures 3D,E). Thus, these data suggest that e-cigarette can impair the ability to fight infection in the lungs, particularly viral infections.

### JUUL Mint Aerosols Induce ACE2 Expression

**FIGURE 4:**
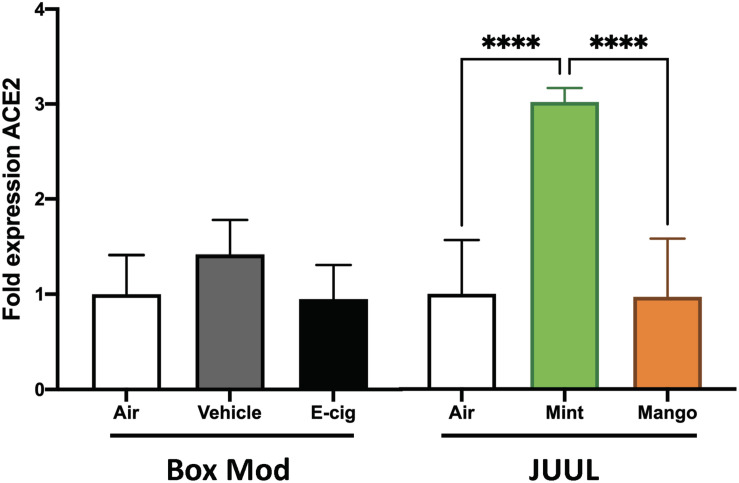
JUUL Mint aerosol inhalation drives upregulation of ACE2 expression within the lungs. Relative gene expression by qPCR from lung tissues of mice exposed for 1 h daily to e-cig aerosols was analyzed. Male C57BL/6 mice were exposed to nicotine containing (Ecig) and no nicotine (Vehicle) e-cig aerosols generated by box Mods (70:30 PG:VG with 6 mg/mL nicotine) for 3 months, with no significant differences found relative to Air controls. Female C57BL/6 mice exposed for 20 min three times daily for 1 month to aerosols generated from JUUL (30:70 PG:Gly with 59 mg/mL nicotine) Mint flavored pods had three-fold greater ACE2 expression in the lung, while chronic JUUL Mango aerosol inhalation did not alter ACE2 expression. ****p < 0.0001. Data are representative of five mice per group.

JUUL were the most popular devices on the market from 2017 until 2020, and their e-liquids are composed of different chemicals than vape pen and box Mod e-liquids, including nicotinic salts at elevated concentrations (69 mg/ml nicotine), benzoic acid, flavorants, and a flipped ratio of PG:Gly of 30:70. Mice exposed to JUUL mint aerosols daily for 3 months developed a 6.1-fold increase in ACE2 expression in their lungs (*p* < 0.0001; Figure 4). Inhalation of aerosols from JUUL mango did not alter ACE2 expression. Aerosols generated by box Mods, with and without nicotine (EV and vehicle, respectively) did not alter ACE2 expression. These data suggest that chemical flavorants in the JUUL mint e-liquid may be involved in the upregulation of ACE2.

## Discussion

The question of how e-cigarette use impacts health has come to the forefront since the 2019–2020 EVALI epidemic in the United States (Bozier et al., 2020). Now, with the COVID-19 pandemic and its related lung damage (ARDS and ALI), the potential associated risk of smoking and vaping at increasing the susceptibility to develop a life-threatening SARS-CoV-2 infection has brought up the interest of researchers in the field (Dai et al., 2020; McAlinden et al., 2020; Soule et al., 2020; The Lancet Respiratory Medicine, 2020).

Some research groups have looked at overall changes induced by e-cigarette aerosols by transcriptomics studies, most of them using *in vitro/ex vivo* human primary cell culture models, which include human nasal epithelial cells (Banerjee et al., 2017; Park et al., 2019), human airway/bronchial epithelia cells (Shin and Crotty Alexander, 2016; Crotty Alexander et al., 2018; Park et al., 2019; Herr et al., 2020), human buccal/oral epithelial (Iskandar et al., 2019; Tommasi et al., 2019), and even cell lines such as human bronchial epithelial BEAS-2B cells (Renegar et al., 2004; Anthérieu et al., 2017). However, to the best of our knowledge, only one study has utilized a rodent model, specifically Sprague-Dawley rat lungs (Phillips et al., 2017). Very few differences in gene expression were found, in contrary to the studies on human cells, although this study had significant conflicts of interest as it was funded solely by Philip Morris International (PMI), and all authors were employees of PMI (Phillips et al., 2017). Thus, our study may be the first to present an unbiased transcriptomic analysis of murine lung tissue as a model, which may provide significant insights as to the broad changes in gene expression of whole lung tissue occurring in response to daily e-cigarette aerosol exposure *in vivo* over time (model of chronic vaping).

In our work presented here, we found that chronic exposure (6 months) to e-cigarette aerosols generated by vape pens induced profound changes in gene regulation within the lungs of both C57BL/6 and CD1 mice. Remarkably, 2,933 gene expression changes occurred in C57BL/6 mice and 2,818 gene expression changes in CD1 mice (Figure 1A). A volcano plot revealed several genes of interest (Figure 1B). This included reduction in IgA in CD1 mice, which is important in lung mucosal immunity (Pilette et al., 2001; Gould et al., 2017). IgA has been found to protect mice against viral respiratory infections (Renegar et al., 2004; Muramatsu et al., 2014; Gould et al., 2017), and its deficiency correlates with airway inflammation and progression of chronic obstructive pulmonary disease (COPD) (Polosukhin et al., 2011). Moreover, other genes were significantly reduced in mice exposed to e-cigarette aerosol, such as CD177 (highly upregulated in patients with severe influenza infection) (Tang et al., 2019), fcmr (which can regulate early B cell activation and plasma cell development after influenza virus infection) (Nguyen et al., 1950), tlr9 (important for viral sensing and correlated with lower respiratory viral loads) (Fulton et al., 2010; Lee et al., 2013), fcgr1 (which contribute to the antiviral-specific antibody responses) (Teijaro et al., 2010; Job et al., 2019), and ccr2 (which promotes viral clearance by enhancing virus-specific T cell responses) (Pamer, 2009; Strunz et al., 2019). In addition, CD4 was also reduced in CD1 but not C57BL/6 mice, which are also important for antiviral responses in the lung (Fulton et al., 2010; Teijaro et al., 2010). Finally, the most upregulated gene in both strains of mice was Krt8, an uncommon keratin gene expressed in epithelial cells. Krt8 has been found to have increased expression in some carcinomas (Chu and Weiss, 2002; Gires et al., 2006) and to promote tumor progression and metastases in gastric carcinoma in particular (Fang et al., 2017), but it has also been involved in alveolar epithelial progenitors in lung regeneration and increased replication of respiratory syncytial virus (Shirato et al., 2012; Strunz et al., 2019).

The modulation of the inflammatory status of the lungs observed in the transcriptomic analysis was associated with impairment of key immune and inflammatory pathways defined by KEGG enrichment plots. On one hand, we found downregulation of pathways involved in cytokine receptor interactions (Figure 1D), chemokine signaling (Figure 1E), and primary immunodeficiency (Figure 1F). On the other hand, we found upregulation of pathways related to cell activation in neutrophils (Figures 1G,H) and dendritic cells (Figure 1I). Activation of neutrophils speeds their demise, by neutrophil extracellular trap formation (NETosis) and apoptosis, such that activation may not be paired with increased numbers of neutrophils if chemokines such as IL-8 are not released. In the particular case of increased activation of neutrophils, it has been shown that prolonged activation of these cells can lead to detrimental effects to the host and can even cause severe disease, including pneumonia and ARDS (Abraham, 2003; Galani and Andreakos, 2015), with ARDS being the main clinical feature of both EVALI (Alexander et al., 2020; Wang J. et al., 2020) and COVID-19 (Matthay et al., 2020; Xu et al., 2020). More recently, it has been shown that severity of COVID-19 is associated with increased activation of neutrophils (Leppkes et al., 2020; Radermecker et al., 2020; Wang J. et al., 2020; Zuo et al., 2020). Thus, this analysis suggests that e-cigarette exposure can affect crucial inflammatory pathways involved in host defense and immune-mediated tissue damage.

In addition, we assessed abundance in the BALF fluid of eicosanoid lipid inflammatory mediators and found decreases in prostaglandin E2 (PGE2) and 12-hydroxyeicosatetraenoic acid (12-HETE), with a trend in 15-hydroxyeicosapentaenoic acid (15-HEPE) (Figure 2). 12-HETE is the major product of 12/15-LOX (lipoxygenases) in rodents and is also generated by a dedicated 12-LOX (which has been reported to be upregulated in the lungs of hypoxic rats) (Preston et al., 2006; Sagliani et al., 2013). 12-HETE stimulates proliferation of pulmonary artery smooth muscle cells and have a possible role in the remodeling process in pulmonary hypertension (Legler et al., 2010; Sagliani et al., 2013). In the case of 15-HEPE, it has been shown to be able to dampen allergic rhinitis symptoms through increased production by eosinophils, leading to inhibition of mast cell degranulation (Sawane et al., 2019; Petrilli et al., 2020). In the context of PEG2, it is the most abundant eicosanoid and a very potent lipid mediator key in many biological functions, such as regulation of immune responses, blood pressure, gastrointestinal integrity, and fertility (Legler et al., 2010; Grewal et al., 2013). PGE_2_ can thus modulate various steps of inflammation in a context-dependent manner and coordinate the whole process in both proinflammatory and anti-inflammatory directions, contributing to the regulation of the cytokine expression profile, and can act as anti-inflammatory on innate immune cells like neutrophils, monocytes, and NK cells (Legler et al., 2010; Ricciotti and FitzGerald, 2011). Interesting enough, reductions in PGE2, 12-HETE, and 15-HEPE seem to be mediated by vehicle components (without nicotine) since vehicle-treated mice had equal to greater changes as compared with EV mice (with nicotine) (Figure 2). Therefore, besides the variety of inflammatory proteins altered due to e-cigarette aerosol inhalation, there are also changes in important lipid mediators involved in inflammation and pulmonary hypertension.

With all that being said, the current global COVID-19 pandemic has raised concern for those individuals with pre-existing conditions, including those with impaired immune response that could increased susceptibility to SARS-CoV-2 infection. This virus is novel and therefore scientists and clinicians have raced to try to understand factors influencing susceptibility to this infection. Early reports indicated that age was the primary risk factor, with mortality rates of >80% reported for patients >80 years of age in some cohorts (Hoffmann et al., 2020; Petrilli et al., 2020). However, more and more young individuals have developed life-threatening COVID-19, suggesting that other factors may influence susceptibility to life-threatening forms of this disease (Grewal et al., 2013; Lee et al., 2014). Such conditions might be inherited or induced by extrinsic factors. Among these extrinsic factors, the use of vaping devices may play a role and should be further studied.

Using deconvolving bulk RNA-sequencing data, we found increased expression of ACE2 in the lung tissue of vape pen-exposed mice (Figure 3A). ACE2 is a crucial protein for SARS-CoV-2 infectivity (Chen and Kolls, 2013; Hoffmann et al., 2020). In addition, we correlated the abundance of RNA-sequencing data to ACE2 expression and found a correlation of ACE2 with a lower abundance of resting CD4^+^ memory T cells but with a higher abundance of follicular helper T cells (Spearman, *p* < 0.05) (Figure 3C). This indicates that there is impairment in adaptive immune responses that are crucial for many infectious diseases. Memory CD4^+^ T cells provide much more immediate protection and are key for vaccine-mediated immunity (Mahmud et al., 2013; Gray et al., 2018). Follicular helper T cells can drive the differentiation of B cells into antibody-secreting cells and to induce the production of high-affinity class-switched antibody (Gray et al., 2018; Hutloff, 2018). Because we used whole lung tissue for RNA extraction, the source of the increased ACE2 gene expression is unknown. In addition, using GSEA, we found that e-cigarette-induced upregulation of ACE2 is correlated with the downregulation of several immune-associated pathways, including interleukin receptor signaling, IFNγ signaling [necessary for Th1 cells, which are essential for host defense against many pathogens, including viruses such as influenza viruses (Chen and Kolls, 2013; Smith et al., 2020)], IL-2/STAT5 pathway [involved in T regulatory cell differentiation and proliferation (Mahmud et al., 2013; Leung et al., 2020b)], and LPA4 pathways and CD28 co-stimulation [a key molecule for T cell activation (Esensten et al., 2016; Leung et al., 2020a)] (Figures 3D–G). This data seems to be linked with the lower abundance of resting CD4^+^ memory T cells observed in Figure 3C. Notably, ACE2 upregulation in e-cigarette aerosol-exposed mice is associated with increased expression of genes associated with influenza infection (Figures 3D,E). Thus, these data suggest that vape pens can increase the expression of ACE2, which associated with impaired key immune pathways involved in the host ability to fight infection, particularly viral infection such as influenza, and potentially SARS-CoV-2.

Despite of the public concern about the potential role of smoking or vaping in COVID-19, so far just a few studies have shown that such relationship exists (Dai et al., 2020; Leung et al., 2020a,b; McAlinden et al., 2020; Russo et al., 2020; Smith et al., 2020). In the case of smoking, some studies have shown that cigarette smoke and COPD can increase the expression of ACE2 in the respiratory tract (Leung et al., 2020b; Smith et al., 2020) and nicotine has been attributed as a driver to this increased ACE2 expression in smokers (Pintarelli et al., 2019; Leung et al., 2020a; Russo et al., 2020; Wang Q. et al., 2020). In contrast, a recent study concluded that it is in fact tobacco and not nicotine that is driving increase in ACE2 expression (Lee et al., 2020), although another study suggested that α7 nicotinic acetylcholine receptor signaling is involved in inducing ACE2 expression (Wang Q. et al., 2020). In the context of e-cigarettes, it has been found that flavorless e-cigarette does not induce ACE2 expression (Lee et al., 2020). Similar to this, we found that chronic, daily inhalation of flavorless nicotine-containing Mod box e-cigarettes did not induce ACE2 expression by qPCR (although flavorless nicotine-containing vape pens increase 33% ACE2 expression based in RNA-sequencing expression analysis). However, daily inhalation of JUUL mint aerosol (but not JUUL mango) leads to increased expression of ACE2 in lung tissue, suggesting that specific flavorants used to create the mint flavor in JUUL pods are driving the increased expression of ACE2. This is of great concern since there are several thousands of chemicals used to provide flavor to e-cigarettes that might be increasing the risk of developing a severe COVID-19. Future studies dedicated to the effects of different flavorants might give insight into increased expression of ACE2 that may account for susceptibility to SARS-CoV-2 infection in relatively young and healthy individuals.

Limitations of this work include the absence of cellular or tissue-level pathology driven by the gene expression changes identified. Further studies are needed to assess for potential altered susceptibility to viral and bacterial pathogens caused by e-cigarette vaping. Although both female and male mice were exposed to e-cigarette aerosols, direct sex effects could not be distinguished due to differences between exposures. It is critical to drill down on potential temporal effects, effects specific to certain chemicals/flavorants, and sex effects of e-cigarette use. There is a need to identify protein, cellular, tissue level, and physiologic read-outs relevant to gene expression changes identified. By combining data across studies, the e-cigarette research community would be well placed to rapidly identify biologically relevant signals occurring from e-cigarette aerosol inhalation. While nicotine concentrations were different in the vape pen, box Mod, and JUUL exposures, the cotinine levels in the plasma of mice immediately after daily exposures were similar. This is consistent with what is known about these devices, that vape pens have poor delivery of nicotine to the bloodstream, while box Mods and pod devices have highly successful nicotine delivery.

Altogether, these data suggest that chronic inhalation of e-cigarette aerosols will lead to numerous gene expression changes within immune and structural cells of the lung, with an overall pattern of immunosuppression, which may diminish host defenses of both innate and adaptive responses in the lungs and lead to increased susceptibility to infections. Alteration of the immune state of the lung by aerosol inhalation is also very likely to impact healing (pulmonary fibrosis), inflammatory responses (hypersensitivity pneumonia, acute eosinophilic pneumonia, and acute interstitial pneumonia), and regulation of genetically damaged cells (lung cancer). Of particular note, we found that flavorants may induce ACE2 expression, increasing the risk to develop severe COVID-19. Flavorant effects escape from most e-cigarette studies due to the wide variety of chemicals used to provide flavor and to simplify the experimental approaches leaving most of the time only the three main components of e-juices, PG, vegetable glycerin, and nicotine.

In summary, beyond the specific impact of vaping on one pathway, these RNAseq data demonstrate a multitude of changes in gene expression which appear to be higher than those seen with cigarette smoke inhalation. Immune (Pintarelli et al., 2019) and inflammatory pathways from both innate and adaptive responses were found to be impacted by chronic inhalation of e-cigarette aerosols, which again raise concern for altered susceptibility to lung infections and inflammatory diseases in human users of these vaping devices. Further studies are needed to define the roles of e-device composition, wattage, chemicals within e-cigarette aerosols (flavorants, vehicles, nicotine, and contaminants), and puff topography on gene expression changes in the lungs.

## Data Availability Statement

The raw data supporting the conclusions of this article will be made available by the authors, without undue reservation.

## Ethics Statement

The animal study was reviewed and approved by UCSD and VASDHS IACUC.

## Author Contributions

LC, SS, WO, and SC: conception and design of the experiments. AM, JM-S, JO, SN, IA, CB, SC, WL, WO, SS, and LC: acquisition, analysis, and interpretation of data. JM-S and LC: manuscript composition. All authors reviewed, contributed to, and approved the manuscript.

## Conflict of Interest

The authors declare that the research was conducted in the absence of any commercial or financial relationships that could be construed as a potential conflict of interest.

## Footnotes

1 https://www.pathwaycommons.org/

2 cibersortx.stanford.edu
